# Discussion and Comments on K_OP_ and ∆K_eff_

**DOI:** 10.3390/ma13214959

**Published:** 2020-11-04

**Authors:** Daniel Kujawski

**Affiliations:** Department of Mechanical and Aerospace Engineering, Western Michigan University, Kalamazoo, MI 49008, USA; daniel.kujawski@wmich.edu

**Keywords:** K_op_, K_eff_, fatigue crack growth, two-parameter crack driving force

## Abstract

This article addresses online discussions with comments related to K_op_ and ∆K_eff_ used in fatigue crack growth (FCG) analyses and modeling. The author of this article assembled an online discussion pertaining to the critical issues and challenges on Kop and ∆K_eff_, which took place during the summer of 2020. The meetings were titled, *Recent Advances on FCG Investigations and Modeling*.

## 1. Introduction

Due to COVID-19 spreading worldwide in the summer 2020, many universities were locked-down and international conferences were canceled. The author of this article organized online meetings using WebEx. The intension was to provide the platform to share and exchange research ideas and their latest results in the area of fatigue and fatigue crack growth (FCG) research. Fatigue researchers from 12 countries participated in this virtual forum including: M. Chapetti (Argentina); K. Walker (Australia); R. Pippan (Austria); J. Castro, M. Meggiolaro (Brazil); G. Glinka (Canada); J. González (Columbia); J. Pokluda, P. Pokorny, T. Vojtek (Czech Republic); R. Heikki (Finland); S.K. Albert, N. Babu, V. Jayaram, A. Kulkarini, M. Mohan, R. Prakash, D.K. Raut, V. Saxena, P. Surajit, R. Sunder (India); P. Strzelecki (Poland); F. Antunes (Portugal); R. Chandran, A. Fatemi, R. Goyal, D. Kujawski, D. Lingenfelser, S. Narasimhachary, J. Newman, Jr., A. Rosenberg, K. Sadananda, A. Saxena, A.K. Vasudevan (USA). The first meeting was held on 9 May 2020, and eight subsequent meetings were held till the end of September. The main theme of these meeting was *Recent Advances on FCG Investigations and Modeling*. The mission of this forum was to generate discussion, debate, and comprehension of different views on FCG with a purpose to expand the understanding on this topic. The goal was to improve existing models/approaches, share insights, and create fruitful discussions on new ideas. This article presents one of the vigorous online discussion on K_OP_ and ∆K_eff_, which took place just before meeting #8, held on 26 September 2020. Comments were assembled in the order they were posted online. All comments were approved and accepted by the participated researchers who posted their comments. 

## 2. Background

Understanding and modeling of fatigue crack growth (FCG) rate is a prerequisite for safe life predictions of components in service. In the 1960s, Paris and Erdogan [1] proposed to corelate FCG rate (da/dN), in terms of an applied ∆K in the form of

(1) $$\frac{\mathrm{da}}{\mathrm{dN}}=C{\left(∆K\right)}^{m}$$

where C, m are fitting parameters.

In such an analysis, each R-ratio forms a discreet (da/dN) vs. ΔK curve. From a practical viewpoint, it is convenient to collapse various R-ratio data into a single FCG rate curve. The first attempt to collapse FCG data for a different R-ratios was proposed by Elber [2] in 1970, who postulated an effective stress intensity factor (SIF) range ΔK_eff_ defined as:

ΔK_eff_ = K_max_ − K_OP_ (or K_CL_) (2)

where K_OP_ is the SIF (due to full crack opening) when there is no contact in the crack wake during loading. Commonly, the K_OP_ term is used interchangeably with K_CL_, where K_CL_ corresponds to the first contact in the crack wake during unloading. It is then inferred that only the single parameter driving force ∆K_eff_ is sufficient to analyze FCG behavior in the form of

(3) $$\frac{\mathrm{da}}{\mathrm{dN}}=C^{*}{\left(∆K_{\mathrm{eff}}\right)}^{m^{*}}$$

where C* and m* are fitting parameters.

While this assumption became an accepted method of analysis for the last 50 years, it omitted the fact that FCG is governed by two SIF parameters: a range ∆K (= K_max_ − K_min_) and K_max_. This insight was brought about 25 years ago by Sadananda and Vasudevan [3,4] when they stated that Equation (1) is valid only for R = 0 where ∆K = K_max_. They advocated that at both thresholds, ∆K_th_ and K_max,th_ must be satisfied simultaneously for a crack to propagate. If only one of them is satisfied, the crack would arrest and not propagate. Thus, for crack extension to occur, ∆K > ∆K_th_ and K_max_ > K_max,th_ must be applied. This dependence gives a L-shape curve for a given da/dN=constant in terms of K_max_ and ∆K signifying that they are interrelated [3,4]. Thus, there are two approaches to the understanding and modeling of FCG phenomenon.

These two approaches were discussed and debated during the online meetings. This article provides an assembled discussion and comments, which took place during the 8th meeting that ended on 26 September 2020.

## 3. Online Discussion and Comments Related to K_op_ and ∆K_eff_

These discussions started when Dr. Tomas Vojtek (Czech Republic) raised the following issue.

“Suppose constant-amplitude loading at the load ratio R = 0. The crack opening level K_op_ is somewhere around 0.25 of K_max_ and it is larger than K_min_. Now I increase K_min_ but I still keep it below K_op_, meaning that the load ratio increased from R = 0 to R = 0.1 or even to R = 0.2. 

Why K_op_ level changes? If there is no material damage and no significant plastic deformation of the crack tip occurs below the K_op_ level, how can K_min_ influence any of the processes leading to a change in the plasticity-induced crack closure? I thought that K_op_ was fully depended on K_max_. But it is larger for minimum K_min_.”

*Prof. Jaroslav Pokluda (Czech Republic):* “I think that, due to imperfections of the crack-face and crack-front closure below the K_op_ (or K_cl_) level, the opening level K_op_ is slightly dependent on K_min_ because of different compressive residual stresses (different level of cyclic plasticity) after unloading to different K_min_.

Therefore, unloading to K_min_ = 0 at R = 0 means a bit higher extent of reversed plasticity (compressive residual stresses) than unloading to a higher K_min_ at R = 0.2. So, if you first load to K_max_ and unload to K_min_ at R = 0 and then load to K_max_ and unload to K_min_ at R = 0.2, the crack opens at a bit higher K_op_ than it was after unloading to K_min_ = 0.”

*Dr. Kuntimaddi Sadananda (USA)*: “My suggestion is to completely ignore plasticity induced closure as it not needed to account for the load ratio effects or to predict crack growth rates in a material. The two-parametric nature involving K_max_ and ∆K are fundamental for the fatigue crack growth and are sufficient to account for the material response. “

*Prof. Jim C Newman, Jr. (USA):* “When the crack opening level is 0.25 K_max_, the cracked material is under nearly plane-strain conditions. K_min_ has a large influence on the K_op_ level. The reason that K_op_ increases as the K_min_ value is increased is because the material left in the wake of the growing crack is not deformed as much during unloading and the crack-opening level is higher. Below K_op_, the material at the crack front and wake are at the compressive yield stress of the material, so a lot of reverse plastic deformation has occurred during unloading below K_op_. But the stress at the crack front is compressive until the crack-front opens. Then the high stress concentration causes the crack-front damage.

You can simulate this behavior with the FASTRAN code that you now have. Give it a try and let me know.”

*Mr. Daniel Lingenfelser (USA):* “This is a good question for us to consider. However, I disagree with your assumption that there is ‘no material damage and no significant plastic deformation of the crack tip occurs below the K_op_ level’. I agree that most of the ‘damage’ occurs above K_op_ but plastic yielding occurs in compression around the crack/notch tip during the unloading part of the cycle. This plastic deformation when the crack is closing determines the K_op_ for the next cycle, Therefore, changing K_min_ will cause a change to K_op_.”

*Prof. Jim C Newman, Jr. (USA):* “Very good reply on the mechanics around a crack front. A lot of plastic deformation occurs during unloading, but there is no plastic deformation upon loading until the crack-tip opens.”

*Prof. Reinhard Pippan (Austria):* “Plasticity induced crack closure is caused by wake plastic zone. There is a monotonic and a cyclic wake plastic zone, the cyclic wake plastic zone reduces the plasticity induced closure induced from the monotonic wake plastic zone. For a constant K_max_ an increase of K_min_ reduces the size of the cyclic wake plastic zone, therefore the closure or better opening load changes with R.” 

*Prof. Ravi Chandran (USA):* “Dan/Jim: I cannot see how crack tip ‘plastic’ deformation, occurring at K < K_op_, can influence crack tip behavior especially when the plastic field of influence (the extent of plastic zone), corresponding to K < K_op_ (at R~0.25 per Tomas), if any, will be far less (the plastic zone will be ≤ one-sixteenth in size relative to MPZS, because PZS scales as square of K). 

I should also point out that any notion of crack tip deformation at K < K_op_ fundamentally violates the definition of ∆K_eff_. For the definition ∆K_eff_ = K_max_ − K_op_ to be fracture mechanically valid, the crack tip should be ‘free’ from any mechanical deformation, regardless of it being elastic or plastic. The formal definition of ∆K_eff_, this way, says that crack tip does not experience ‘any’ crack tip field for K < K_op_. 

However, this definition itself at serious fault (because of K_op_ entering in the definition) since, in actual experiments, net-section is actually under non-zero elastic stress for K < K_op_, thus invalidating the definition. I pointed out this difficulty in one of my earliest posts in this group. 

To accept crack closure as a crack tip ‘shielding effect’ one should experimentally demonstrate that the crack tip and the remaining ligament is absolutely at zero stress, until K_op_ is reached in loading. I do not see how can one demonstrate this, especially when the specimen is to be loaded mechanically to reach that K_op_, which means the net-section will have to be loaded to the full stress field corresponding to the state of K_op_. This is the principal reason that Jaime and others find elastic field activity in the crack tip region at K < K_op_.

I can’t see how we can circumvent this simple argument. I look forward to any comments from all, if I am not seeing what others are seeing.”

*Prof. Grzegorz Glinka (Canada)*: “Very good question! You have received already several explanations and I would also add a bit of spice to it. 

Fatigue crack growth is controlled by the stress and strain field ahead of the crack tip. Both the maximum stress/strain and the stress/strain range are important. However, according to the most popular SWT fatigue damage parameter it is sufficient to use only the maximum stress and the strain range in the form of the product of the maximum stress and strain range or σ_max_Δε. This product can be translated into the equivalent fatigue crack growth parameter in the form of ${\left(∆K\right)}^{1-p}{\left(K_{\max}\right)}^{p}\;$ and subsequently into the Walker parameter ${\left(∆K\right)}^{0.5}{\left(K_{\max}\right)}^{0.5}\;$ in the case when the power is *p* = 0.5. It indicates that the fatigue process whether analyzed within the nominal stress theory S-N, the local stress–strain theory ε-N or the fracture mechanics da/dN-ΔK, depends on two loading/stress parameters, i.e., the maximum and the range. Therefore, if any of the two parameters gets changed the fatigue crack growth rate will change as well because it depends on the resultant combination of the two of them. 

Crack tip stresses and strains and the resultant driving force depend (but in a highly nonlinear fashion) on both K_max_ and ΔK. The third factor outside of the applied K_max_ and ΔK is the residual stress σ_r_ generated by the cyclic plasticity ahead of the crack tip. The plasticity induced residual stresses exist ahead of the crack tip regardless whether the crack tip is closed or not and whether the crack surface behind the crack tip is smooth or rough. The plasticity induced residual stresses just ahead of the crack tip are COMPRESSIVE or zero! Crack tip closure is not required for the existence of compressive residual stresses ahead of the crack tip! Therefore the fatigue crack growth modelling based on the crack tip closure (i.e., the displacements field behind the crack tip) might be incapable of capturing certain load history effects occurring in situations where there is no closure or (even more critical) when the entire crack is closed as in cases when significant part of the loading cycle is in compression. Consistent analysis of stress and strain ahead of the crack tip under the compressive part of the load/stress cycle shows visible effect of the applied compressive load on the variation of crack tip stress/strain field. The closure model might not be capable of modelling such cases unless we assume that even under total compression of the cracked body the crack tip stays open. 

Therefore, I am biased towards fatigue crack growth models based on the analysis of the elastic-plastic stress–strain response of the material ahead of the crack tip and the closure phenomenon should come out as a part of the solution but not as an imposed phenomenon.”

*Prof. Daniel Kujawski (USA)*: “Pertinent to this discussion are the experimentally based conclusions from the following paper: Fatigue Crack Closure: A Myth or A Misconception? J. Tong, S. Alshammrei, B. Lin, T. Wigger, J. Marrow Fatigue & Fracture of Engineering Materials Structures, 2019, vol. 42, pp. 2747–2763.

Main conclusion from the above paper: Both visual observation and compliance curves were used to determine the ‘crack opening’ levels; whilst the impacts of the crack opening on the crack driving force J and the normal strains ahead of the crack tip were evaluated in 2D and 3D. The results from the study indicate that, crack closure, although clearly identifiable in the compliance curves, does not appear to impact on global crack driving force, such as J-integral, or strains ahead of the crack tip, hence it may well be a misconception.”

*Prof. Jaroslav Pokluda (Czech Republic)*: “I think that we are in agreement that the key factor is the extent of the cyclic plastic deformation that reduces the ‘thickness’ of the crack-wake wedge (responsible for the plasticity-induced crack closure) when decreasing the K_min_. The only thing I would like to stress is that the dependence of the K_op_ (or K_cl_) on the K_min_ at the constant K_max_ is rather weak, because of the absence of the stress concentration behind the crack front.”

*Prof. Jaime PT Castro (Brazil)*: “I like very much Prof. Pippan’s explanation, but it contradicts the central point in Elber’s argument. If ‘there is no activity ahead of the crack tip below K_op_’, how come the cyclic plastic zone (pz_c_) can change K_op_ when K_min_ increases but remains below the original K_op_ value? Moreover, if pz_c_ can affect or even control K_op_, then pz_c_ would not be the main cause for (supposed) K_op_ effects? 

However, instead of pursuing such an argument line, let me recall that the main idea behind Julián’s thesis, whose results motivated our exciting Saturday meetings (González, JAO et al. Challenging the ‘ΔK_eff_ is the driving force for fatigue crack growth’ hypothesis. Int. J. Fatigue 136:105577, 2020), was to experimentally **verify** questions like that. When we decided to make very simple FCG tests under constant {∆K, K_max_} conditions, measuring the opening load along the entire crack path in thin and thick DC(T) and C(T) steel and Al specimens, we were also (although indirectly) testing if K_op_ was being controlled by pz_c_. However, without any load interaction effects (we even designed a system to continuously adjust the loads as the crack grew, to avoid such undesirable effects), we clearly proved that while the FCG rates remained constant along the entire crack path (thus indeed showed no retardation effects), K_op_ significantly decreased along the entire crack path. Hence, ∆K_eff_ = K_max_ − K_op_ was NOT controlling the FCG rates in all those tests. Since we measured K_op_ by all accepted methods (at least when they are used to support Elberian arguments), and since all of them yielded similar results, I cannot see why we still are trying to explain how Tomas’ question does not contradicts ∆K_eff_ hypotheses. 

However, since Elber’s ideas seem to have achieved a dogma status, let me propose a new simple challenge: based on our meetings discussions, we should at least agree once for all that discussions about ∆K_eff_ arguments must be based on proper K_op_ measurements.”

*Dr. Ramasubbu, Sunder (India)*: “I woke up to a mail trail that reflects on how open a subject closure is:

1. Tomas’s question (as usual) sets one thinking—one may consider the following possibilities:
  a. Crack closure is not an event. It is a process that starts along the specimen surface and concludes at the mid-section. It is quite possible that at mid-thickness, particularly in thicker materials, the crack simply will not close—perhaps, even under compression. Therefore, localized crack-tip inelastic response to applied K between Kop and Kmin cannot be ruled out.
  b. Crack-tip stress–strain response is likely to see dramatic variations along the crack front. This is a 3D problem. LEFM suggests that rate of change of local stress with K (dσ/dK) is a constant at any point ahead of the crack-tip. This cannot be. It is indeed possibly determined almost entirely by applied K while K > K_cl_. But once wake contact takes place at any point on the vast real estate behind the crack tip, it is no longer the case—at any point ahead of the crack tip!
  c. A realistic model of crack-tip cyclic stress–strain response must consider non-linear elastic crack-tip response associated with crack closure—that must be treated as always partial, never complete. Strictly speaking, this will also affect how local mean stress affects growth rate if cumulative damage concepts are applied—there is empirical evidence of growth rate turning cycle-sequence insensitive in the event of partial crack closure.
2. To add to Reinhard’s comment—cyclic inelastic crack wake response must be out of the question because the wake can only yield in compression. But cyclic elastic wake response, indeed, is likely to be affected by cyclic inelastic response ahead of the crack tip because residual strain due to the latter will most certainly affect wake contact stress—both axially as well as by hinge effect. FASTRAN possibly models this effect.
3. To Ravi’s comment—The crack tip, like all notches seeing cyclic inelastic response will never see ‘absolutely unloaded’ condition. Closure as a phenomenon is not interpreted to imply unloading of the crack tip, and indeed, seizure of crack tip response is not synonymous with an unloaded crack tip. A simple rationale behind this interpretation is the universal agreement that crack tip stress–strain field at K_max_ is unaffected by closure, that in turn leads to the conclusion that closure ‘clips’ the lower end of crack tip response—allows little further change in crack-tip stress strain state once applied K drops below K_cl_.”

*Prof. Daniel Kujawski (USA)*: “Most of the colleagues suggest that K_min_ affects K_op_ (or K_cl_). Providing that ∆K_eff_ = K_max_ − K_op_ represents a crack driving force, it means, that ∆K_eff_ is affected by K_min_ through it influence on K_op_. Thus, the crack driving force is dependent on both K_max_ and Kmin (a two-parameter driving force). Do we need necessary K_op_ in FCG analyses?”

*Prof. Marco A Meggiolaro (Brazil)*: “Dan Lingenfelser, thank you for the input. So, if a lower K_min_ mitigates a former closure effect caused by K_max_, then naturally you would need to later load beyond K_op_ (perhaps until the same K_max_) to recover the original higher closure level. 

Now consider a loading history ranging only between K_min_ and the current K_op_, never beyond K_op_, while K_min_ is slowly increased (never decreased). Closure theory would require K_op_ not to increase at all, and of course the crack not to grow at all. If on the other hand, K_op_ ends up changing in this decreasing-applied-∆K case, then some cyclic plastic process during both loading and unloading must be causing it; thus there should be cyclic damage below K_op_ and eventually crack growth, against Elber’s hypothesis. This is a simple discriminant experiment if we can all agree (beforehand) on a proper K_op_ measurement method (the updated K_op_ could be measured in the end of the experiment, since it might require loading beyond the original K_op_ used in the applied cycles; or we could use DIC techniques to continuously identify the opening level at least in thin specimens, like we have done using several redundant and agreeing techniques to properly obtain K_op_.

To spice things up, we could design the experiment to start with a high steady-state K_op_ value such that ideally K_op_ >> K_max_,_th_ and (K_op_ − K_min_) >> ∆K_th_ from the Universal Approach. Different specimen thicknesses could also be used to better evaluate the actual evolution of K_op_. Any thoughts about this scenario? (K_min_-to-K_op_ ranges with slowly increasing K_min_ and high original K_op_).

I am looking forward to the next WebEx meeting. Prof. Newman’s results intrigue me. How come is the calculated crack opening stress zero for a high-R baseline loading, with an abrupt rise after four 25% overloads, which then does not decay after the loading returns to its original baseline value? And all this happening with a growing crack, not an arrested one. I would really like to see measurements of such opening loads, to make sure they indeed happen. 

Despite FASTRAN’s good results, both Math (crack growth rates) and Physics (K_op_ measurements) must agree if closure is to be validated.

Finally, thank you all and especially Dan Kujawski for these valuable discussions, they bring back good memories reminding me of the essence of the early Hyannis conferences.”

**Figure 1:**
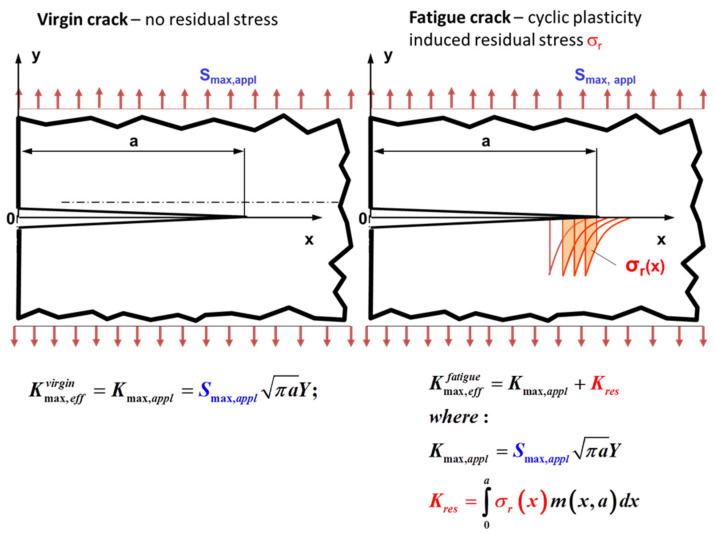
An illustration of plasticity induced residual stresses.

*Prof. Grzegorz Glinka (Canada)*: “There is a lot emphasis put on the crack tip closure and the effective stress/SIF range. I would like to mention that residual stresses created by the crack tip cyclic plasticity have noticeable effect on the effective maximum stress intensity factor as well. The figure below shows two identical cracked components subjected to the same applied maximum stress S_max,appl_. One component (on the left) contains virgin crack without any stress history and having zero stress at S_appl_ = 0 (beginning of the loading process). The stress intensity factor K^virgin^_max,eff_ at the nominal stress level S_appl_ = S_max,appl_ is shown below. However, in the case of a propagating fatigue crack, there is residual stress field created by the cyclic loading when propagating the crack up to its length ‘a’. This residual stress σ_r_ exists even when the applied stress is S_appl_ = 0. The applied maximum cyclic stress is must be superposed on the pre-existing residual stress σ_r_. Therefore, the local stress field ahead of the virgin crack will be different than the stress field ahead of the fatigue crack (right hand side of Figure 1). As a result the two effective stress intensity factors induced by the same applied maximum stress S_max,appl_ must be different as well, K^virgin^_max,eff_ ≠ K^fatigue^_max,eff_.

Therefore residual stresses induced by the cyclic plasticity around the fatigue crack tip (left hand side of Figure 1) reduce (because they are compressive) both the applied maximum stress intensity factor (K_max,appl_) and the applied stress intensity range (ΔK_appl_). It appears that this effect is stronger than the crack tip closure (if occurs) appearing as suggested at the end of the descending reversal. This is the reason why the UniGrow concept is based on the superposition of K_max,appl_ and ΔK_appl_ and K_res_. 

It is also worth to add that the displacement fields (COD behind the crack tip) are also different at the maximum load S_max,appl_, indicating that the maximum stress intensity factor is affected by the residual stress as well. I am not questioning the existence or nonexistence of the crack tip closure but I am not sure whether concentrating fatigue crack models solely on variations of the minimum stress intensity factor is sufficient and consistent from the point of view of the behaviour of the crack tip stress/strain field.”

*Dr. Kuntimaddi Sadananda (USA):* “Greg, you have brought up a very important point. Residual stresses are a subset of internal stresses which affect both crack initiation and growth. This is particularly important in the following cases:

1. Crack initiation and growth in the short crack growth regime.
2. Crack growth under overloads and underloads.
3. Crack initiation near preexisting stress concentrations.
4. Crack growth in TRIP (transformation induced plasticity) steels, etc.

Crack closure invokes a ‘similitude breakdown in the short crack growth’ regime. 

From our point, there is no similitude breakdown. Cracks in a virgin sample get initiated after 10^7^ cycles or so, which creates dislocation pile-ups, intrusions, and extrusions, etc., which are stress concentrations formed by fatigue damage. Cracks initiate due to these local stress concentrations, which are needed to be accounted for in the short crack initiation and growth. As Kitagawa-Takahashi have shown even if one takes a preexisting short crack, many cycles are needed for internal stresses to build up before the crack moves forward. 

As Kramer (1974) has shown (I.R. Kramer, A Mechanism of Fatigue Failure, Metallurgical Transaction, Vol. 5, 1974, 1735–1742.), if one electropolishes the specimen that removes fatigue damage near the surface, the life can be extended significantly.

These are experimental facts. 

Since the internal stresses are localized, their effect goes down as the crack grows and moves out of their range. The same concept applies to underloads and overloads, TRIP steels, etc. This is a Unified Concept. We recently wrote a review paper on short crack growth. 

In essence, no need to invoke crack closure or lack of it or similitude breakdowns, etc. Fracture mechanics is valid across the board. I just could not resist writing after seeing Greg’s post.”

*Prof. Ravi Chandran (USA)*: “My point was that as long as we use the definition of ∆K_eff_ = K_max_ − K_op_, we strictly imply that the crack tip is fracture mechanically unloaded at K < K_op_. I do not see any way around this in the process of calculations of ∆K_eff_, with opening occurring as an event at K = K_op_.

To me, it seems that the ‘seizure’ of crack tip and ‘clipping’ of the lower end of crack tip response is no different from saying K = 0 until K = K_op_ on loading. 

Do you mean to suggest that there is a different form of ∆K_eff_ other than that given by ∆K_eff_ = K_max_ − K_op_ with the occurrence of K_op_ as an event?”

*Prof. Jaime TP Castro (Brazil)*: “I do agree with your (Ravi’s) opinion. Elber’s **hypothesis** that the FCG driving force is ∆K_eff_ = K_max_ − K_op_ implies in no activity whatsoever ahead of the crack tip for K < K_op_ loads. Everything else is just a filibuster to avoid recognizing that Elber’s hypothesis may not be true in all cases. Recall that besides ours plenty of constant da/dN data measured at fixed {∆K, K_max_} loading conditions, but with a huge variation of ∆K_eff_ along the crack path, we showed as well through direct DIC measurements strain variations below K_op_, and claimed that such results cannot be explained by Elberian arguments. See González, JAO et al. Challenging the ‘ΔK_eff_ is the driving force for fatigue crack growth’ hypothesis. Int. J. Fatigue 136:105577, 2020 for the data in question. Changes in da/dN measured at fixed K_max_ and K_op_ but with variable K_min_ < K_op_ cannot be explained by those arguments either. This does not mean that Elber’s ∆K_eff_ cannot be the FCG driving force in some other conditions, but it certainly is **not** in such cases. Hence, it is **not** a universal model for FCG.” 

*Prof. Jim C Newman (USA)*: “See the attached picture. You have to look at both the loading and unloading behavior at the crack tip to understand the complete picture. 

**Figure 2:**
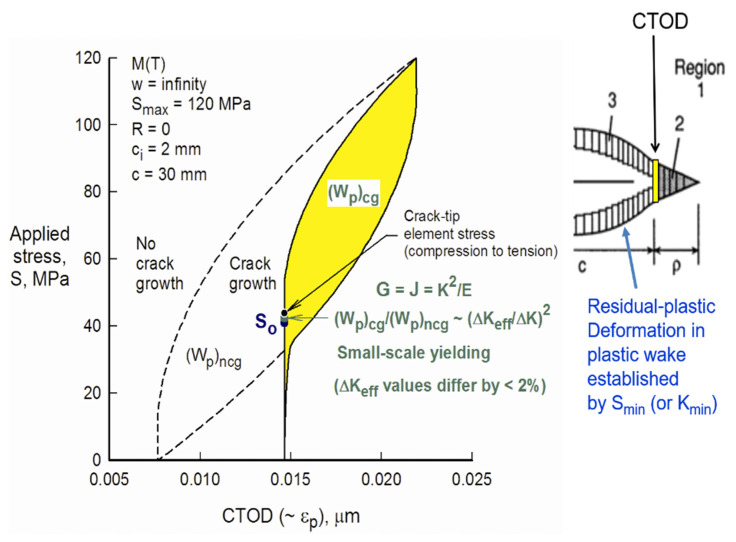
FASTRAN simulation of the crack tip deformation.

On the loading cycle, the crack-tip material has ‘no’ plasticity deformation until the crack tip opens (crack tip has large stress/strain concentration). (CTOD is only plastic deformation and is related to the cyclic plastic-strain range, which has been affected by crack closure.) Thus, above the crack-opening stress, the material begins to have plastic deformation. During the loading process, the crack grows by an increment ∆c (dc/dN per cycle). At K_max_, the increment ∆c has a large amount of plastic deformation and the growth increment (element) closes at a very high applied stress. (The yellow element in Figure 2 has now broken and becomes residual plastic deformation along the crack surface.) 

During unloading, the growth increment goes into reverse plastic deformation until the bulk plastic wake closes and keeps the crack-growth increment from further plastic deformation. Thus, K_min_ is very important in establishing the contact stress field. From the contact stress field, the new crack-opening stress for the next loading cycle is calculated.”

*Dr. Ramasubbu Sunder (India)*: “My dear Ravi: My feedback was with specific reference to your comment ‘To accept crack closure as a crack tip “shielding effect” one should experimentally demonstrate that crack tip and the remaining ligament is absolutely at zero stress, until K_op_ is reached in loading.’” 

*Dr. Pavel Pokorný (Czech Republic)*: “Dear professor Newman: I am afraid that it is still not clear to me why loading cycles with various K_min_ below K_op_, but with the same K_max_ lead to the change in K_op_ (based on picture you have attached in your previous message). Please could you explain more your idea? Regarding to picture attached in your last message I would like to kindly ask you if redistribution of stress is considered after yellow strip breakage? I mean that if yellow element (the first element in plastic zone) is broken at maximal applied stress then stress field is redistributed even before unloading. Is that included in FASTRAN?”

*Prof. Jim C Newman, Jr. (USA)*: “Dear Pavel: Just call me ‘Jim’. And thanks for asking me to clarify my comments. 

When the crack-tip element (yellow) breaks, the stress field is redistributed before unloading. Also, we need to talk about K_min_ < K_cl_ and not K_op_. During unloading, the broken element will contact at a very high applied stress (K_cl_) and reverse yield until more of the crack surface contacts. This prevents the amount of reverse plastic deformation on the broken (yellow) element. There is another closure load when the bulk of the plastic wake contacts—this value is close to the opening load, K_op_. Thus, the length of the residual plastic deformation elements is set by the repeated applications of K_min_ (or S_min_).

The slide also shows that the strain range (cyclic CTOD) in the crack-tip region has been greatly influenced by crack-surface contact. Thus, crack closure has to be considered when calculating the crack-front stress and strain fields.

The FASTRAN model includes residual stresses in the plastic zone under cyclic loading. Thus, the K_op_ value is influenced by the residual-stress field in the forward plastic zone. But, FASTRAN uses ∆K_eff_ and not residual stresses to calculate damage, but residual stresses are very important. Note that ∆K_eff_ is a function of ∆K and R (or ∆K and K_max_); and thus, the concept includes the two crack-driving parameters.”

*Prof. Reinhard Pippan (Austria)*: “Dear Pavel: Closure is an effect of loading history, and not only of the considered single load cycle. In a constant load amplitude experiment in fracture mechanic experiment the d∆K/da is usually small, therefore this history effect disappears. But if you start with a closure free crack than you can clearly see this history effect. You could then also see the difference in cyclic plastic zone at the same K_max_ but different positive K_min_. 

Here a very old result of a constant ∆K experiment at different R ratio, on very sharp notches where at the beginning no crack closure takes place even not in compression (Fatigue Fract, Engin Mater. Stuct 1987, 319; or Metallurgical Transaction 18A, 1987, 433).”

## 4. Conclusions

It is clear from the above discussion that despite more than 50 years devoted to crack closure research, its measurement, simulation, and their effects on FCG, there seems to be no common agreement among the researchers. It is up to the new generation of researchers and more precise and accurate experimental techniques to clarify the issue on crack closure or whether a single or two-parameter driving force is more suitable for FCG analyses. 

## References

[1] Paris P., Erdogan F. (1963). A Critical Analysis of Crack Propagation Laws. J. Basic Eng..

[2] Elber W. (1971). The Significance of Fatigue Crack Closure. Damage Tolerance in Aircraft Structures.

[3] Vasudevan A.K., Sadananda K., Louat N. (1993). Two critical stress intensities for threshold fatigue crack propagation. Scr. Metall. Mater..

[4] Vasudevan A.K., Sadananda K., Louat N. (1994). A review of crack closure, fatigue crack threshold and related phenomena. Mater. Sci. Eng..

